# Cloning and Functional Analysis of *ClVND1*, a Member of the OsNAC7 Subfamily of the NAC Family in *Chrysanthemum lavandulifolium*

**DOI:** 10.3390/plants14182925

**Published:** 2025-09-20

**Authors:** Yueyue Liu, Chendi Mei, Hao Zhang, Ying Liao, Yinuo Zhai, Hai Wang, Xuebin Song

**Affiliations:** 1 College of Landscape Architecture and Forestry, Qingdao Agricultural University, Qingdao 266109, China; 20232235005@stu.qau.edu.cn (Y.L.); mcd0724@126.com (C.M.); 20242135037@stu.qau.edu.cn (H.Z.); 20242135050@stu.qau.edu.cn (Y.L.); 20242135006@stu.qau.edu.cn (Y.Z.); 2 Shandong Key Laboratory for Germplasm Innovation of Saline-Alkaline Tolerant Grasses and Trees, Qingdao Agricultural University, Qingdao 250000, China

**Keywords:** NAC, *ClVND1*, Stress, *Chrysanthemum lavandulifolium*

## Abstract

*Chrysanthemum × morifolium* is a commercially important flower worldwide. *Chrysanthemum lavandulifolium* is the main model plant for the research on *Chrysanthemum*. Enhancing stress resistance in *C. lavandulifolium* is highly significant for improving commercial *chrysanthemum* production. *NAC* transcription factors are key regulators of plant growth, development, and stress responses. In this study, we cloned *ClVND1*—a member of the OsNAC7 subfamily within the NAC transcription factor family—from *Chrysanthemum lavandulifolium*. The gene comprises a 1164 bp coding sequence (CDS) encoding a protein of 387 amino acids. Overexpression of *ClVND1* promotes secondary cell wall thickening in the stems of transgenic *Arabidopsis*, stimulates lateral root growth, and consequently enhances tolerance to salt and low-temperature stress in seedlings. Phenotypic analysis showed that transgenic *Arabidopsis* exhibited reduced inflorescence elongation and plant height compared to wild-type controls, but an earlier flowering time. These findings suggest that *ClVND1* enhances stress resistance by promoting lateral root development, while also suppressing inflorescence growth and accelerating flowering time.

## 1. Introduction

During growth and development, plants are frequently exposed to a range of abiotic and biotic stresses, including drought, high salinity, low temperature, and pathogen infection. These stresses typically induce cellular damage, adversely impacting plant growth and productivity [1]. Transcription factors play important roles in plant adaptation to the environment and resistance to stress. NAC transcription factors play a key role in regulating secondary wall synthesis and serve as major regulators of broad-spectrum stress resistance in plants [2,3]. Within the NAC family, the OsNAC7 subfamily is one of the most extensively studied groups and includes key members such as *SND1*, *NST1*, *URP7*, *BRN1/2*, and *VND1*–*VND7* [4,5,6,7,8,9,10,11,12,13]. Its primary functions involve the regulation of secondary cell wall formation in stems, roots, and anthers. They play a key role in regulating lignin biosynthesis, growth and development, as well as stress responses [11,14,15,16,17]. Within the OsNAC7 subfamily, *NST1*, *NST2*, and *SND1* are classified as secondary wall-associated NAC transcription factors and function as master switches that activate the expression of genes involved in secondary cell wall biosynthesis [18]. Previous studies have demonstrated that the *ClSND1* gene from *Chrysanthemum nankingense* promotes thickening of the secondary xylem and its cell walls in transgenic *Nicotiana benthamiana*, thereby conferring enhanced tolerance to salt and low-temperature stress in seedlings. Additionally, it suppresses plant growth and promotes early flowering [1,19,20]. Recent studies have revealed that NAC transcription factors are expressed throughout plant development and across various tissues, where they play crucial roles in lignin biosynthesis, growth regulation, and responses to both abiotic and biotic stresses [21]. A total of 102 NAC family members have been identified in pepper (*Capsicum annuum*) [22], 101 in tomato (*Solanum lycopersicum*) [23], 190 in maize (*Zea mays*) [24], 143 in grape (*Vitis vinifera*) [25], and 152 in soybean (*Glycine max*) [26]. Overexpression of the *ZmNAC071* gene in *Arabidopsis* enhances plant stress tolerance, while the celery *AgNAC63* gene is highly responsive to various abiotic stresses [27,28].

VND is a group of NAC family transcription factors that encode a specific NAC domain. Seven vascular-related *VND* genes have been found in *A. thaliana*. Zhou et al. investigated the roles of *VND1*–*VND5* genes in regulating secondary cell wall biosynthesis in *Arabidopsis thaliana* [29,30]. *VND1*–*VND5* expression was specifically observed in vessel elements, with *VND4* and *VND5* also detected in the secondary xylem vessels of the root hypocotyl [11]. Tan et al. demonstrated that *VND1*, *VND2*, and *VND3* play distinct roles in the differentiation of xylem vessel elements within cotyledons. The VND subfamily has also been examined in banana (*Musa*), in addition to well-established models such as *Arabidopsis* and *poplar*. *MusaVND1* encodes a highly conserved NAC domain and is a homolog of *Arabidopsis VND1* [31]. *MusaVND1* activates the expression of genes involved in the lignin and cellulose biosynthetic pathways. Transgenic banana plants overexpressing *MusaVND1* exhibited increased lignin and cellulose content compared to wild-type controls [32]. Previous studies indicate that the five *VND* genes in *Chrysanthemum nankingense* participate in regulating root and stem development, as well as mediating responses to drought and salinity stress [33]. However, the functional role of *VND* genes in *C. lavandulifolium* has not yet been reported [34].

## 2. Results

### 2.1. Bioinformatic Characterization of the ClVND1 Gene

A gene designated *ClVND1* was cloned from *C. lavandulifolium*. Its coding region comprises 1164 base pairs, which encode a polypeptide of 387 amino acids. The *ClVND1* gene sequence was obtained from the NCBI database (accession no. PX275817). We found that the *ClVND1* gene sequence shares high sequence similarity with *Chrysanthemum nankingense* CHR0003673-RA (Appendix A).

The physicochemical properties of the ClVND1-encoded protein were evaluated. The physicochemical analysis revealed that this protein is composed of 351 amino acid residues. Its theoretical isoelectric point (pI) was calculated to be 5.31, with a relative molecular mass of 40,897.95 Da. The molecular formula was predicted as C_1814_H_2788_N_492_O_558_S_15_ (total atoms: 5667), and its instability index was 69.15. The protein has an average hydrophobicity of −0.672 and an instability index (II) of 45.37, indicating that it is unstable. ProtScale analysis revealed that the ClVND1 protein possesses distinct hydrophobic and hydrophilic regions, and was overall determined to be hydrophilic. Analysis of the ClVND1 protein sequence predicted the absence of transmembrane domains, indicating a non-transmembrane structure (Appendix B).

**Figure 1:**
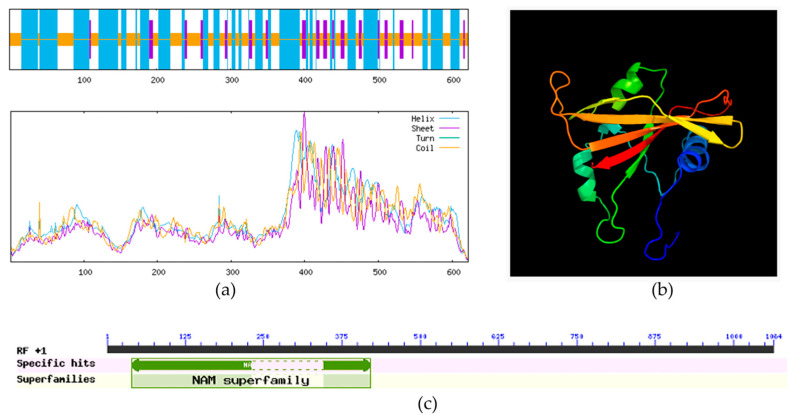
Bioinformatic Characterization of the *ClVND1* gene in *Chrysanthemum lavandulifolium*. (**a**) Prediction of Protein Secondary Structure. (**b**) Prediction of Protein Tertiary Structure. The helical structures represent alpha-helices of the protein, the folded forms represent beta-sheets, and the loop regions do not possess a clearly defined regular structure, serving as flexible areas that connect different secondary structural domains. (**c**) Identification and Analysis of the Conserved Domain in the *ClVND1* Gene.

The secondary structure of the ClVND1 protein was predicted to consist primarily of irregular coil (43.09%) and α-helix (46.62%), with extended strands accounting for the remaining 10.29% (Figure 1). The tertiary structure of the ClVND1 protein was predicted using Phyre2 and was found to be consistent with its secondary structure prediction (Figure 1). The function and domain architecture of the ClVND1 protein were predicted using the CD-Search online tool. Results indicated that the protein belongs to the NAC family (Figure 1). NAC transcription factors, which are unique to terrestrial plants, play pivotal roles in regulating plant growth and development, hormonal signal transduction, and stress responses. The *ClVND1* gene has been implicated in mediating plant responses to adverse environmental stresses.

**Figure 2:**
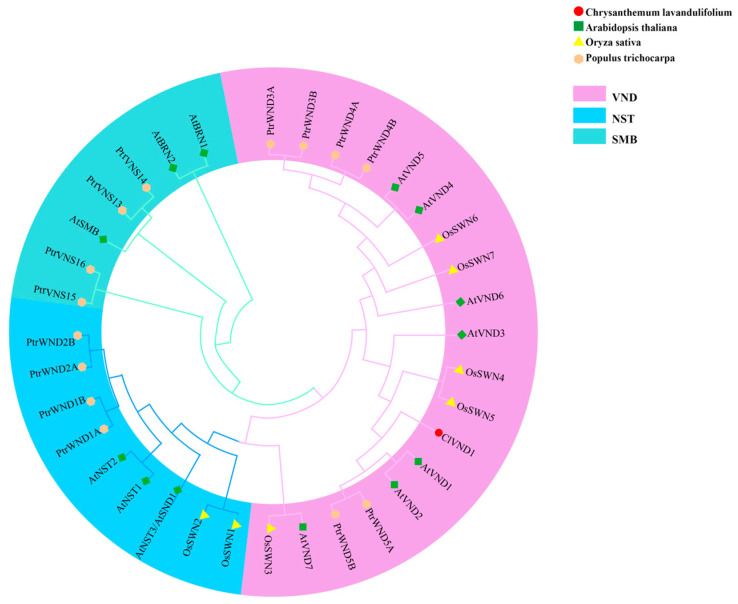
Phylogenetic Analysis of ClVND1 among VNS Proteins from Divergent Taxa.

The evolutionary relationships among VNS proteins were inferred by constructing a maximum likelihood phylogenetic tree using amino acid sequences from diverse species. Phylogenetic analysis indicated that ClVND1 is most closely related to AtVND1, AtVND2, PtrWND5A, and PtrWND5B, and most distantly related to AtVND7 and OsSWN3 (Figure 2). ClVND1 was phylogenetically grouped within the same clade as homologs from *Arabidopsis thaliana* (AtVND1, AtVND2, AtVND3), *Oryza sativa* (OsSWN6, OsSWN7), and *Populus trichocarpa* (PtrWND5A, PtrWND5B). Based on these phylogenetic findings, ClVND1 can be classified as a member of the VND subfamily.

### 2.2. Analysis of Subcellular Localization in Nicotiana benthamiana

**Figure 3:**
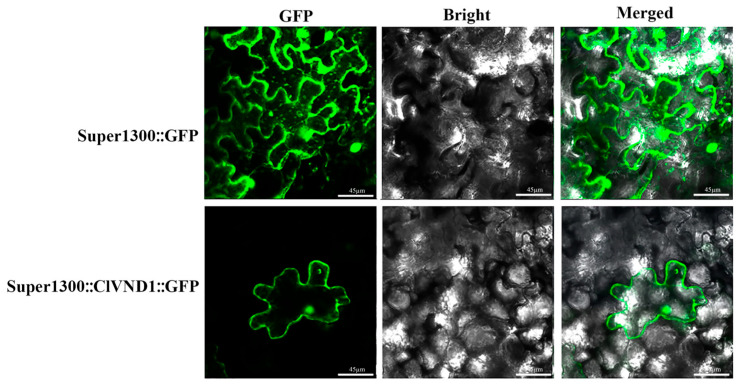
Analysis of Subcellular Localization in *Nicotiana benthamiana*. Green fluorescence typically refers to the light emitted by Green Fluorescent Protein (GFP).

To determine the subcellular localization of the ClVND1 protein, fluorescence-based localization analysis was performed in transgenic *Nicotiana benthamiana*. The fluorescence signal in the leaves of the transgenic plants was observed, and it was found that the Super1300 empty transgenic *N. benthamiana* could recognize the fluorescence signal in the nucleus and cell membrane of the leaves. Fluorescence signals were detected in the nucleus and cell membrane of Super35S::*ClVND1*::GFP transgenic *N. benthamiana* leaves (Figure 3). These results indicate that *ClVND1* is localized in the nucleus and cell membrane.

### 2.3. Identification of Positive Transgenic Lines and Validation by qRT-PCR

**Figure 4:**
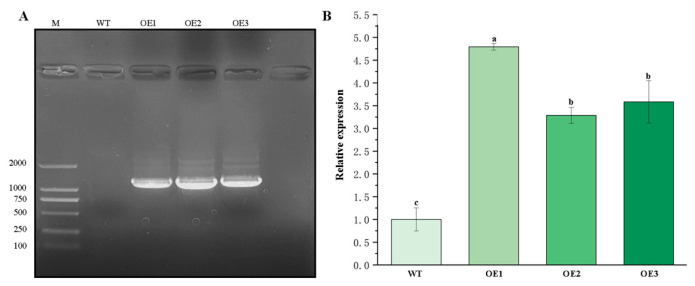
(**A**) Identification of positive transgenic plants by PCR. WT: Wild-type; OE1, OE2, OE3: Transgenic lines. “M” denotes the super DNA marker. (**B**) Real-time PCR. WT: Wild-type; OE1, OE2, OE3: Transgenic lines. The different letters are significantly different (*p* < 0.05). Bars indicate standard errors (n = 3).

Genomic DNA was isolated from healthy wild-type (WT) and transgenic *Arabidopsis* lines to serve as a template for PCR with primers 1300-F/1300-R, using WT plants as a negative control. Amplification of a specific 1164 bp fragment was observed exclusively in the transgenic lines (Figure 4A). The absence of this band in the WT control confirms the successful genomic integration of the *ClVND1* transgene, thereby validating the transgenic plants at the DNA level. To examine the expression level of *ClVND1* in each transgenic line, cDNA reverse-transcribed from both wild-type (WT) and transgenic plants was used as the template for amplification with gene-specific primers *ClVND1*-F/*ClVND1*-R and the reference primers Actin8-F/Actin8-R. As shown in Figure 4B, compared to the WT, the expression levels in all OE-*ClVND1* lines were significantly increased. Among them, the OE-1 line exhibited the highest expression level, which was 4.79 times that of the WT, while the OE-2 line showed the lowest expression level, which was 3.28 times that of the WT. Based on its highest expression level, the OE-1 line was selected for subsequent experiments.

### 2.4. Response of Transgenic A. thaliana to NaCl and Low-Temperature (4 °C) Stress Treatments

**Figure 5:**
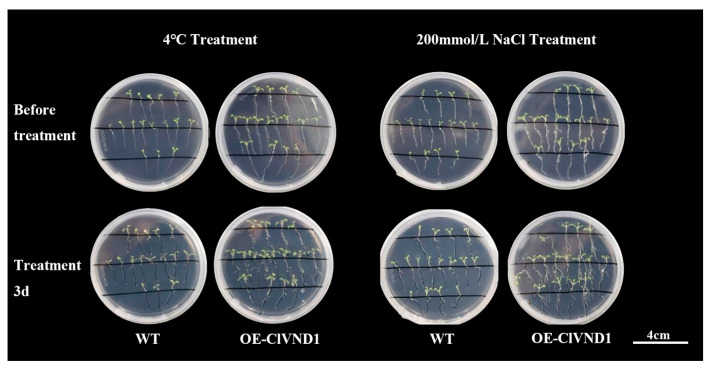
Growth of *Arabidopsis thaliana* under 200 mmol/L NaCl and 4 °C treatments for 3 days.

Under 4 °C conditions over a three-day period, the root upper part of *ClVND1*-transgenic *A. thaliana* displayed significantly enhanced growth relative to wild-type controls, suggesting improved cold stress tolerance. Under 200 mmol/L NaCl, *CLVND1*-overexpressing lines exhibited a developed substantially more of short lateral roots compared with wild-type plants (Figure 5).

**Figure 6:**
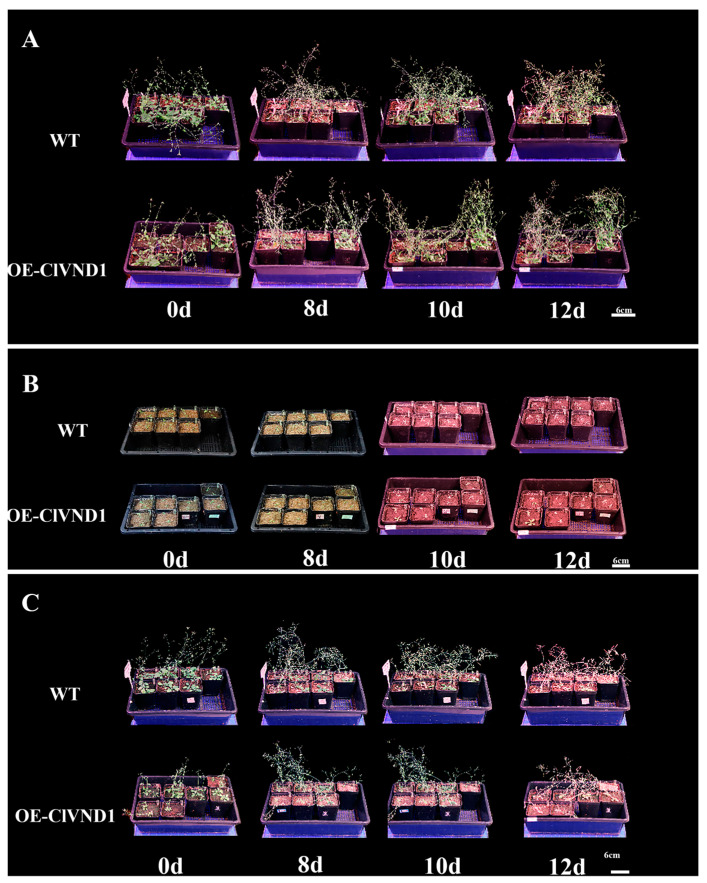
Phenotype of *Arabidopsis* under 4 °C and NaCl treatments. (**A**) Untreated *A. thaliana.* (**B**) *A. thaliana* under 4 °C low-temperature stress. (**C**) *A. thaliana* treated with 200 mmol/L NaCl.

**Figure 7:**
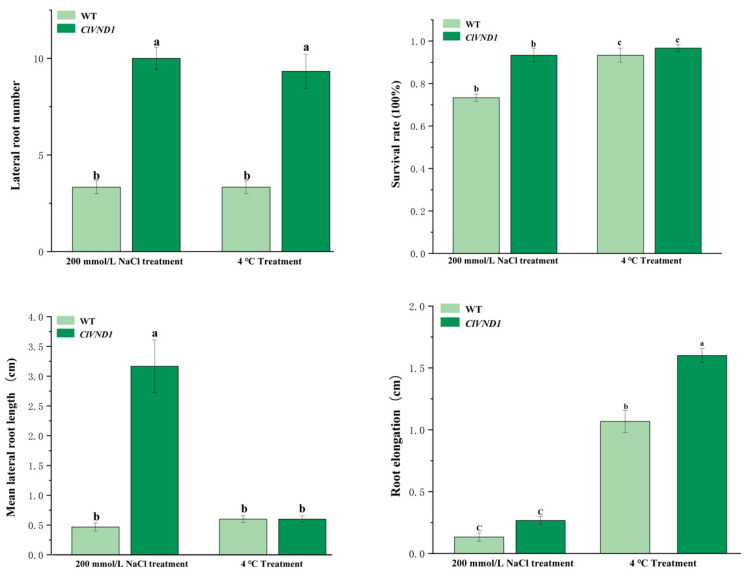
Growth status statistics of *A. thaliana* at 4 °C and NaCl treatments for 3 days. The different letters are significantly different (*p* < 0.05). Bars indicate standard errors (n = 3).

After phenotypic comparison between experimental and control groups, quantitative metrics were collected and subjected to statistical analysis (Figure 6). Under NaCl stress, *ClVND1*-overexpressing *Arabidopsis thaliana* exhibited enhanced lateral root development, showing significantly greater lateral root number and length compared to wild-type controls. Transgenic plants produced an average of 10.0 lateral roots versus 3.33 in wild-type plants (*p* < 0.05). The lateral root length in transgenic lines was approximately 100% greater than that of wild-type specimens, and their survival rate was 27% higher under stress conditions. Under 4 °C treatment, *ClVND1*-overexpressing transgenic *Arabidopsis thaliana* showed a pronounced increase in lateral root production relative to wild-type controls. The average lateral root length in transgenic plants was 13% of that in wild-type specimens, while lateral root extension reached only 50% of the wild-type level. Furthermore, the survival rate of transgenic lines was 4% of that observed in wild-type plants (Figure 7). The results demonstrated that *ClVND1*-overexpressing *Arabidopsis thaliana* exhibited enhanced lateral root growth under stress conditions compared to wild-type plants, indicating that the *ClVND1* gene from *Chrysanthemum lavandulifolium* plays a crucial regulatory role in root development under abiotic stress.

### 2.5. Observation of the Growth and Development of Transgenic A. thaliana

**Figure 8:**
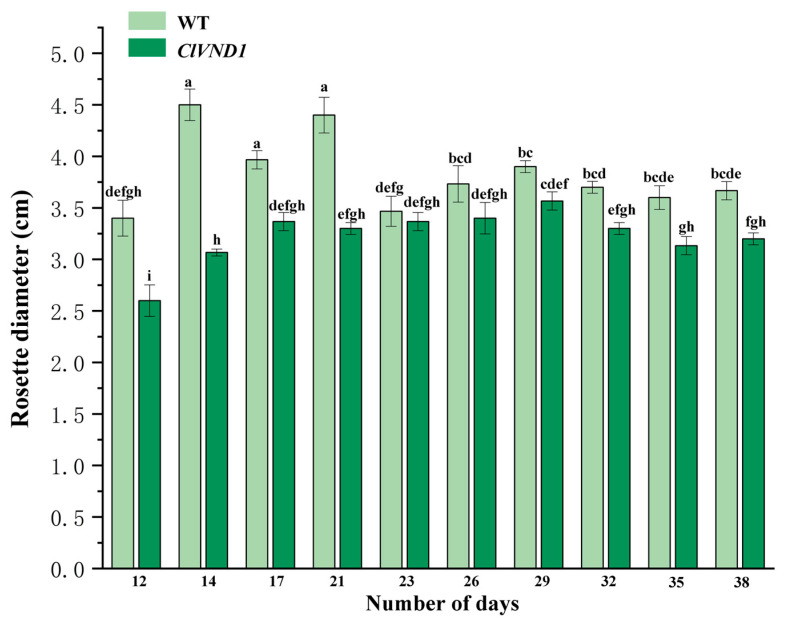
Rosette Diameter of Transgenic *A. thaliana*. The different letters are significantly different (*p* < 0.05). Bars indicate standard errors (n = 3).

We transplanted transgenic and wild-type *A. thaliana* seedlings into 7 cm × 7 cm pots after they had developed two true leaves. The rosette diameter was measured and statistically analyzed, with the results presented in Figure 8. The results indicated that during the early growth stage, wild-type *Arabidopsis thaliana* exhibited a larger rosette diameter compared to transgenic lines, suggesting that the vegetative growth of the transgenic *Arabidopsis thaliana* was inhibited.

**Figure 9:**
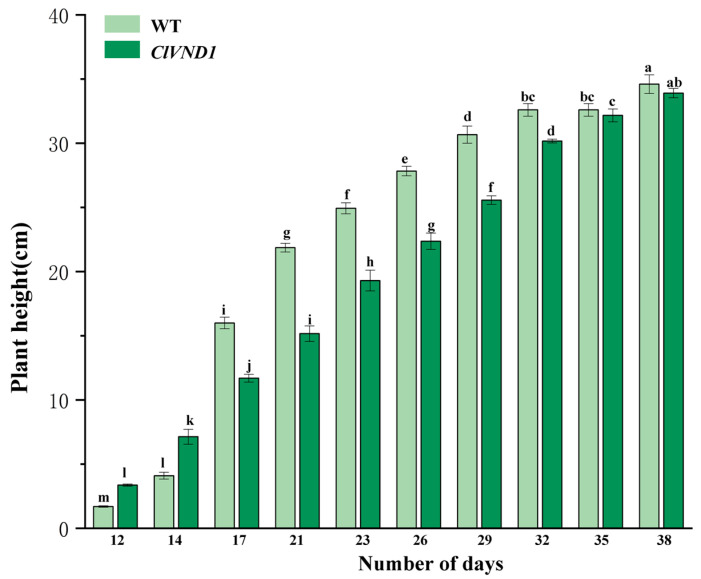
Growth height statistics of transgenic *A. thaliana*. The different letters are significantly different (*p* < 0.05). Bars indicate standard errors (n = 3).

Analysis of the statistical results of *Arabidopsis thaliana* height (Figure 9) revealed that with the growth of *Arabidopsis thaliana* inflorescences, the elongation rate and height of wild-type *Arabidopsis thaliana* inflorescences were higher than those of transgenic *Arabidopsis thaliana*, indicating that the growth of transgenic *Arabidopsis thaliana* inflorescences was also inhibited. Before the 17th day, the plant height of the transgenic *Arabidopsis thaliana* was higher than that of the wild-type *Arabidopsis thaliana*. After the 17th day, the plant heights of both wild-type and transgenic plants showed an upward trend. However, the plant heights of transgenic *Arabidopsis thaliana* were all lower than those of wild-type plants, indicating that the growth and development of transgenic *Arabidopsis thaliana* were restricted. However, the flowering period of the transgenic materials was earlier than that of the control group. Wild-type *Arabidopsis thaliana* began to produce inflorescences 9 to 12 days after transplantation and started to flower on the 12th day. *Arabidopsis thaliana* transformed with the *ClVND1* gene began to produce inflorescences 7 to 12 days after transplantation and started to flower on the 10th day. Based on the above experimental results, it is concluded that *ClVND1* resists salt stress and low temperature stress by regulating the root system, while inhibiting the growth of inflorescences and promoting flowering.

### 2.6. Paraffin Section Analysis of Stem Segments in Wild-Type and Transgenic Arabidopsis thaliana

**Figure 10:**
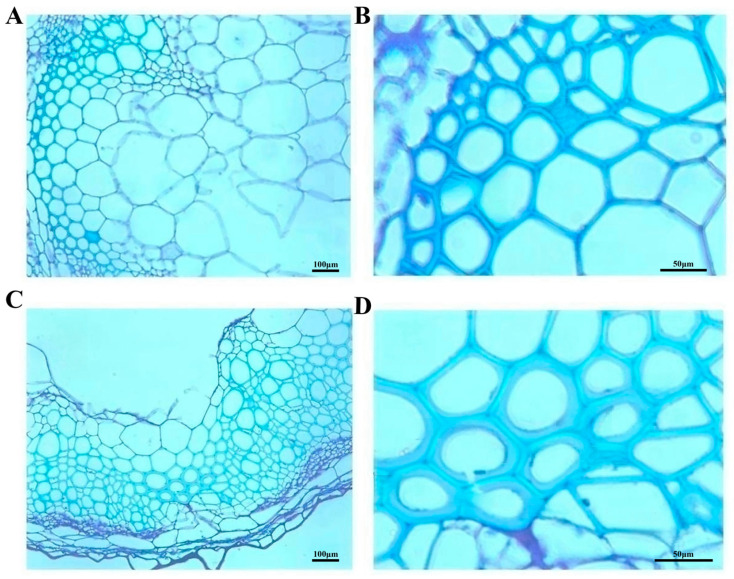
Stem Anatomy of 1300::GFP and *ClVND1*-Transgenic *A. thaliana*. (**A**,**B**) Wild-type *A. thaliana* stem cross-sections. (**C**,**D**) *ClVND1*-transgenic *A. thaliana* stem cross-sections.

Paraffin sections of entire stem segments (3–4 cm in length) from 1300::*ClVND1*::GFP and 1300::GFP transgenic *A. thaliana* were prepared and examined. As shown in Figure 10, paraffin sections of stem tissues from 1300::*ClVND1*::GFP and 1300::GFP (control) transgenic *A. thaliana* were examined. Morphometric analysis revealed a significant increase in secondary cell wall thickness in *ClVND1*-overexpressing plants compared to the control. The mean secondary cell wall thickness was 11.631 μm in 1300::*ClVND1*::GFP transgenic lines versus 4.85 μm in 1300::GFP control lines, demonstrating that *ClVND1* overexpression enhances secondary cell wall deposition (Figure 10).

## 3. Discussion

As an important ornamental flower, the exploration of drought-resistant related genes and the selection and breeding of low-temperature and salt-tolerant *Chrysanthemum lavandulifolium* through molecular biological methods provide new approaches for the growth and development of modern *Chrysanthemums* and molecular breeding for stress regulation. NAC family transcription factors have been demonstrated to confer broad-spectrum stress resistance in plants. The research results demonstrate that the *ClVND1* gene enhances plant tolerance to both salt and cold stress. This finding aligns with previous reports on other members of the NAC transcription factor subfamily. The OsNAC7 subfamily is one of the most extensively studied subfamilies within the NAC transcription factor family, and includes key members such as *SND1*, *NST1*, *URP7*, *BRN1/2* and *VND1*–*VND7*. Previous studies have demonstrated that the *ClSND1*-like gene contributes to lignin biosynthesis in *Chrysanthemum lavandulifolium* and participates in the regulation of plant growth, salt tolerance, and osmotic stress response [35]. *ClBRN1* overexpression significantly thickens secondary cell walls in *Arabidopsis thaliana*, thereby enhancing plant mechanical strength and stress resistance [36]. The *ClNUM1* gene plays a role in the synthesis of secondary cell walls, thereby enhancing the resistance of *Nicotiana benthamiana* [37]. These findings are consistent with the aforementioned study. Furthermore, it was observed that transgenic lines exhibited a higher average number of lateral roots along with an earlier flowering time compared to wild-type plants. In 2019, Yang et al. employed an *Arabidopsis*-based overexpression vector to functionally characterize the soybean NAC gene *GmNAC109* [38]. Sequence analysis revealed that *GmNAC109* shares high homology with *ATAF1*, a regulator of plant responses to both biotic and abiotic stresses. Overexpression of *GmNAC109* enhances abiotic stress tolerance and promotes lateral root formation. Furthermore, transgenic plants exhibited earlier flowering compared to wild-type plants. Zhang et al. overexpressed the *CcNAC1* gene in jute (*Corchorus capsularis*) to investigate its biological function [39]. The relative expression level of the 3-ketoacyl-CoA synthase (*KCS*) gene was significantly up-regulated in transgenic plants, which also exhibited earlier flowering compared to wild-type controls [40]. This finding aligns with the results of the present study. Olsen et al. identified the NAC-preferred cis-elements CGT[GA] and their extensions TTNCGTA and TTGCGTGT, and revealed that NAC proteins bind these motifs as dimers. This discovery establishes the molecular foundation for elucidating *ClVND1*’s DNA-binding specificity [41].

Paraffin sections were made from stem segments of transgenic and wild-type *Arabidopsis thaliana* for observation, and it was found that the thickness of secondary cell walls in the stems of transgenic *Arabidopsis thaliana* with *ClVND1* gene was significantly greater than that of the control group. Studies have shown that the eight genes (*PTVNS01*-*08*) homologous to VND in poplar and *Arabidopsis thaliana* play a significant role in xylem development and secondary wall formation. *PtVNS7*/*PtrWND6* and *PtVNS8*/*PtrWND6B*, similar to *Arabidopsis VND7*, exert primary regulatory functions and play a significant role in vessel differentiation and secondary wall thickening [42,43]. It is consistent with the above results.

In this study, we cloned the *ClVND1* gene from *Chrysanthemum lavandulifolium*, which encodes a protein of 351 amino acids. Bioinformatic analysis revealed that *ClVND1* contains a conserved NAC domain and is predicted to be an unstable, non-transmembrane protein, confirming its classification within the NAC family. Subcellular localization indicate that the ClVND1 protein is localized to both the nucleus and the cell membrane. Although ClVND*1* is predicted to be devoid of transmembrane helices, its transient expression consistently reveals dual localization at the plasma membrane and the nucleus. This pattern parallels the well-documented behaviors of *NPR1*, *OsMADS25*, and *bZIP28*—transcriptional regulators that likewise lack canonical transmembrane domains yet accomplish membrane-to-nucleus shuttling via distinct mechanisms: *NPR1* through redox-controlled oligomerization and subsequent disulfide-mediated dissociation [44,45], *OsMADS25* via N-terminal myristoylation and palmitoylation [46], and *bZIP28* via proteolytic liberation of a soluble, NLS-bearing fragment [47]. Consequently, the nuclear–plasma membrane dual localization of ClVND1 is not anomalous; rather, it exemplifies a conserved paradigm in which non-transmembrane plant transcription factors or signaling proteins exploit lipid modification, redox sensitivity, or proteolytic processing to achieve signal-dependent subcellular relocalization.

*ClVND1* is concurrently induced by low temperature and salt stress in *Chrysanthemum lavandulifolium*, positioning it as a pivotal candidate for enhancing stress tolerance in this species.

## 4. Materials and Methods

### 4.1. Experimental Materials

Aseptic seedlings of *Chrysanthemum lavandulifolium* were provided by the College of Landscape Architecture and Forestry at Qingdao Agricultural University and cultured on 1/2MS medium [36]. Genetic transformation of *ClVND1* was conducted in *Arabidopsis thaliana* [36], and its subcellular localization was analyzed using *Nicotiana benthamiana* [36]. All plants were grown in an artificial climate chamber at Qingdao Agricultural University (Qingdao, China) under controlled conditions: temperature of 25 °C, relative humidity of 60%, light intensity of 2500 lx, and a photoperiod of 16 h light/8 h dark.

### 4.2. Test Method

#### 4.2.1. Target Gene Fragment

Leaves of *C. lavandulifolium* with good growth were selected for total RNA extraction. The total RNA extraction kit used was the FastPure^®^ Universal Plant Total RNA Isolation Kit (Vazyme, Nanjing, China). The reverse transcription kit used was the HiScript III 1st Strand cDNA Synthesis Kit (+gDNA wiper) (Vazyme, Nanjing, China). The full-length coding sequence of the target gene was designed according to the *Chrysanthemum nankingense* genome data (Table 1). The full-length coding sequence of the target gene was obtained by high-fidelity DNA polymerase 2 × Phanta Flash Master Mix (Dye Plus) (Vazyme, Nanjing, China). The PCR thermal cycler adopts the T Series Multi-Block Thermal Cycle (LonGene^®^, Hangzhou, China). The PCR amplification system was 25 µL and the reaction system was as follows: 2 × Phanta Max Master Mix: 12.5 µL; *ClVND1*-F: 1 µL; *ClVND1*-R: 1 µL; cDNA: 2 µL; ddH_2_O: 8.5 µL. The reaction procedure was predenaturation at 95 °C for 3 min. Denaturation at 95 °C for 15 s, annealing at 56 °C for 15 s, extension at 72 °C for 60 s, 35 cycles; Extend at 72 °C for 5 min (LonGene^®^PCR thermal cycler, Hangzhou, China). The PCR products were separated by agarose gel electrophoresis. the PCR products were recovered by bands with the same length as the target gene and sent to Sangong BioEngineering (Shanghai, China) Co., Ltd. for sequencing.

#### 4.2.2. Bioinformatics Analysis

Based on the amino acid sequence of *ClVND1*, amino acid sequence multiple alignment was performed using MEGA11.0.13, and its sequence was analyzed in detail. DNAMAN version 8.0 (Lynnon Biosoft, Quebec, QC, Canada) was used to align the sequences.

#### 4.2.3. Observation of the Subcellular Localization Signal

The Super1300::*ClVND1*::GFP overexpression vector was subsequently transformed into *N. benthamiana* by the Agrobacterium-mediated floral dip method, Select *N. benthamiana* leaves that have been infected for 4 weeks and place them in the dark for 2 to 3 days. The GFP signals in the lower epidermis of *N. benthamiana* leaves were observed by fluorescence microscope (Leica DM 2500, Wetzlar, Germany) and laser confocal microscope (Leica TCS SP5, Wetzlar, Germany). The overexpressed Super 1300-GFP were all retained in the laboratory [36].

#### 4.2.4. Construction of the Super1300 Overexpression Vector

The design of primers containing restriction sites is shown in Table 1.

According to the CDS region of the *ClVND1* gene and the multiple cloning sites of pSuper1300::GFP vector, primers containing *Pst*I endonuclease and *Kpn*I endonuclease restriction sites and without a termination codon were designed. The target fragment containing restriction site was obtained by PCR reaction and agarose gel recovery. The target fragment containing the restriction site and pSuper1300::GFP vector were double digested (37 °C, 2 h) with endonucleases *Pst*I and *Kpn*I (NEB, Beijing, China) to obtain the target fragment and linear vector with sticky ends. The recombinant plasmid was transformed into *E. coli* competent DH5α (WEIDI, Shanghai, China) and spread on a solid LB medium containing 50 mg/L kanamycin for resistance screening and identified by colony PCR. The resulting positive plaque was sent to a sequencing company for sequencing and plasmid recovery. The constructed pSuper1300::*ClVND1*::GFP expression vector was transformed into Agrobacterium tumefaciens GV3101 (Shanghai, Weidi Biology, Shanghai, China) by freeze-thawing and spread on the LB solid medium with 3 antibiotics (50 mg/L kanamycin, 50 mg/L gentamicin, 50 mg/L rifampicin) for resistance screening. The positive colonies was identified by colony PCR and transferred to the liquid LB culture of 3-antibiotics for propagation. After these positive strains were sent for sequencing in Sangong BioEngineering (Shanghai, China) Co., Ltd.

#### 4.2.5. Identification of Transgenic Plants

To identify transgenic positive plants, we extracted total DNA from the leaves of healthy wild-type (WT) and transgenic *Arabidopsis* lines, which served as templates for PCR amplification using the 1300-F/1300-R primer pair. The wild-type plant was used as a negative control, allowing molecular identification of transgenic plants at the DNA level. Total DNA was extracted from healthy, well-growing A. thaliana leaves using the FlaPure Plant DNA Extraction Kit (Gensand Biotech, Beijing, China). The target gene was obtained by high-fidelity DNA polymerase 2 × Phanta Flash Master Mix (Dye Plus) (Vazyme, Nanjing, China). The PCR thermal cycler adopts the T Series Multi-Block Thermal Cycle (LonGene^®^, Hangzhou, China). The PCR amplification system was 25 µL and the reaction system was as follows: 2 × Phanta Max Master Mix: 12.5 µL; 1300-F: 1 µL; 1300-R: 1 µL; cDNA: 2 µL; ddH_2_O: 8.5 µL. The reaction procedure was predenaturation at 95 °C for 3 min. Denaturation at 95 °C for 15 s, annealing at 58.2 °C for 15 s, extension at 72 °C for 60 s, 35 cycles; Extend at 72 °C for 5 min (LonGene^®^PCR thermal cycler, Hangzhou, China). DNA fragments were separated by agarose gel electrophoresis and visualized using a DNA marker (GenStar, Beijing, China).

#### 4.2.6. Quantitative Real-Time PCR Assay

RT-qPCR analysis was conducted on wild-type and transgenic plants. Tissue samples of *A. thaliana* were flash-frozen in liquid nitrogen, homogenized into a fine powder, and transferred to RNase-free 2 mL centrifuge tubes. Total RNA was extracted from healthy, well-growing *A. thaliana* leaves using the FastPure^®^ Universal Plant Total RNA Isolation Kit (Vazyme, Nanjing, China). Subsequently, cDNA was synthesized from the extracted RNA using the HiScript III 1st Strand cDNA Synthesis Kit (+gDNA wiper) (Vazyme, Nanjing, China). RT-qPCR was performed on a StepONE Plus system (Applied Biosystems, Waltham, MA, USA) using TB Green^®^ *Premix Ex Taq*^TM^ II (Takara, Shiga Prefecture, Japan) and specific primers (Table 2). Three biological replicates were performed for each reaction, and the results were analyzed using the 2^−ΔΔCT^ method. Data were visualized and statistically analyzed for significance using SPSS 27 and Origin 2022 software.

#### 4.2.7. Transformation and Screening of *A. thaliana*

The Super1300::*ClVND1*::GFP overexpression vector was transformed into *A. thaliana* by the Agrobacterium-mediated floral dip method [48], after which the transgenic *A. thaliana* seeds were screened by a resistance screening medium (1/2MS + 30 g/L sucrose + 6 g/L agarose + 50 mg/L hygromycin) [36].

#### 4.2.8. Stress Treatment and Phenotypic Observation

Abiotic stress treatment was carried out during the seedling stage of transgenic *Arabidopsis thaliana*, and 1/2MS medium was selected for subsequent experiments [36]. The transgenic *Arabidopsis thaliana* plants were subjected to salt stress at 4 °C and 200 mmol/L, and wild-type *Arabidopsis thaliana* grown under normal conditions was used as the control. Three days after processing, photos of *Arabidopsis thaliana* were taken and data were recorded.

Plants of similar growth vigor were selected from each treatment of Super1300::GFP and Super1300::*ClVND1* lines for stress treatment. The salt treatment was conducted by applying a 200 mmol/L NaCl solution. Low temperature treatment using a low temperature incubator was performed at a temperature of ±4 °C with light for 12 h. The two treatments were cultured at ±25 °C, and a normal water supply was used for the control (CK). After 3 days, the transgenic plants of *A. thaliana* were photographed, and related data were collected.

#### 4.2.9. Observation of the Growth and Development of Transgenic *A. thaliana*

Transgenic *A. thaliana* and the control group were transferred into a 7 cm × 7 cm flowerpot when two true leaves had grown. Under consistent growth conditions, rosette diameter, plant height, and flowering time were measured for both groups.

#### 4.2.10. Paraffin Sectioning

Stem segments from the upper one-third of *Arabidopsis thaliana* were collected and fixed in FAA solution (70% alcohol:formalin:acetic acid 18:1:1) for 24 h. The samples were then softened in a 1:1 mixture of hydrogen peroxide and glacial acetic acid for 48 h. Subsequent processing included dehydration through an ethanol series, paraffin embedding, and sectioning at 10 μm thickness using a microtome. Finally, the samples were stained with safranine green. For each experiment, three biological replicates were prepared. The cell wall thickness of ten randomly selected cells per view was measured, and statistical analysis was performed.

#### 4.2.11. Data Analysis

Three biological replicates and technical replicates were performed for treatments in this study. Excel2010 was used to organize the data, SPSS27 was used for statistical analysis, one-way ANOVA and least significant difference method were used to analyze the data (*p* < 0.05), and Origin2022 software was used to plot the data.

## 5. Conclusions

In summary, *ClVND*1 was cloned from *C. lavandulifolium* and identified as a gene of the OSNAC7 subfamily which encodes a nuclear protein and a cell membrane protein. This study found that the *ClVND1* gene can regulate the growth and development of *A. thaliana* as well as improve its stress tolerance, shown by the enhanced tolerance of salt and low temperature, and increase the thickness of the secondary woody cell wall of its stem.

## Figures and Tables

**Table 1:** The design of the primer that contains the enzyme tangent site.

Primer Name	Sequence (5′→3′)	TM/°C	Purpose
*ClVND1*-F	gcTCTAGAatgatcatggacacagtaga	57.29	Amplification of the full-length VND1 sequence with restriction sites.
*ClVND1*-R	aaCTGCAGatcaaaaatgcataatcca	54.71	
*pSuper1300*-F	gtgacgccatttcgccttttc	57.69	
*pSuper1300*-R	ggtggtgcagatgaacttcagg	58.69	

**Table 2:** Primer sequences used for quantitative real-time PCR (qRT-PCR).

Primer Name	Sequence (5′→3′)	TM/°C	Purpose
*ClVND1*-F	GTTTTGGAAGGCTACGGGGA	59.96	Real-time PCR Primers
*ClVND1*-R	GTTTGTCCCGTTGCACGTTT	60.18	
Actin8-F	TCAGCACTTTCCAGCAGATG	55.22	
Actin8-R	CTGTGGACAATGCCTGGAC	56.61	

**Figure A1:**
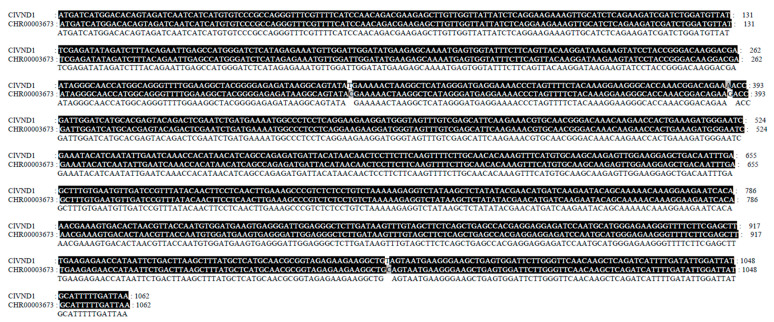
Cloning and analysis of CDS sequences of the *ClVND1* gene.

**Figure A2:**
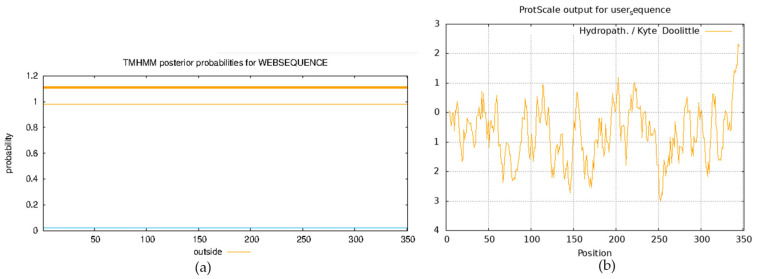
Bioinformatic Characterization of the *ClVND1* gene in *Chrysanthemum lavandulifolium.* (**a**) Analysis of transmembrane domain architecture. (**b**) Evaluation of hydrophilic and hydrophobic characteristics.

## Data Availability

The datasets generated during and/or analysed during the current study are available from the corresponding author on reasonable request.
